# Usmile likelihood evaluation provides robust threshold free assessment of binary classification models for balanced and imbalanced datasets

**DOI:** 10.1038/s41598-026-40545-z

**Published:** 2026-02-20

**Authors:** Barbara Więckowska, Przemysław Guzik

**Affiliations:** 1 Department of Computer Science and Statistics, Poznan University of Medical Sciences, Poznan, Poland; 2 Department of Cardiology-Intensive Therapy and Internal Medicine, Poznan University of Medical Sciences, Poznan, Poland; 3 University Centre for Sports and Medical Studies, Poznan University of Medical Sciences, Poznan, Poland

**Keywords:** Binary classification, Likelihood ratio, Threshold-independent evaluation, Model assessment, Imbalanced data, Variable selection, Explainable machine learning, Medical research, Mathematics and computing

## Abstract

Current metrics for binary classification, like the Area Under the Receiver Operating Characteristic curve (AUC-ROC) or Log Loss, provide a global performance score. However, they do not quantify predictive quality separately for event and non-event classes. This limitation is particularly critical in imbalanced settings like medical diagnostics. To address it, we introduce the U-smile Likelihood Evaluation (LE) method, a substantial extension of the original U-smile framework. The U-smile LE method is based on a new metric called the relative Likelihood Ratio (rLR). This single score measures overall model strength without needing a classification threshold. We decompose this score into two class-specific components: $\:{rLR}_{1}$ for event class and $\:{rLR}_{0}$ for non-event class, visualizing them simultaneously in a compact U-shaped plot. We validated the U-smile LE method on synthetic datasets with varying class imbalance and a real-world clinical Heart Disease dataset. In severely imbalanced scenarios (90/10 class distribution), stepwise variable selection guided by U-smile LE outperformed traditional AUC-based selection, improving minority-class detection by 16% in the Area Under the Precision-Recall curve (AUC-PR) and 21% in F1-score. The evolution of U-smile patterns during variable selection provided clear, interpretable insight into class-specific contributions of individual predictors. Demonstrated with both logistic regression and random forest models, U-smile LE offers an explainable, model-agnostic framework for evaluating binary classifiers, especially valuable where class imbalance and interpretability are key concerns.

**Supplementary Information:**

The online version contains supplementary material available at 10.1038/s41598-026-40545-z.

## Introduction

Binary classification plays a central role in medical diagnostics, risk prediction and machine learning applications. A persistent challenge is evaluating models without relying on arbitrary thresholds, which complicates comparisons across datasets, populations and modeling frameworks^1,2^.

Traditional threshold-free metrics – the Area Under the ROC curve (AUC), Log Loss or the Cost of log likelihood ratio (Cllr) – summarize global performance but do not distinguish predictive quality for event and non-event classes and may behave suboptimally in imbalanced settings^3–6^. This limitation is particularly important in medical applications, where the consequences of false negatives and false positives differ substantially^7–9^.

Growing evidence shows that receiver operating characteristic (ROC) analysis becomes less informative under strong imbalance, Precision-Recall (PR) curves or likelihood-based metrics provide better sensitivity to minority-class performance^5,6,10^ in such cases. However, all these frameworks continue to aggregate information across classes, offering no direct insight into whether performance gains arise from improved detection of event class, non-event class, or both.

To address this gap, we previously introduced.

the U-smile framework – a visualization approach that quantifies how predictors affect model performance separately for each class^11–13^.

In this study, we substantially extend that framework by introducing the U-smile Likelihood Evaluation (LE) method. U-smile LE formalizes the relative Likelihood Ratio (rLR) as a unified, threshold-free metric of predictive strength and decomposes it into class-specific and subclass-specific components. This advances the interpretability of the original U-smile framework while adding a rigorous statistical foundation and significance testing.

Our contributions are threefold:

1. We define the rLR coefficient and establish a complete mathematical framework enabling both class-wise and global model evaluation.
2. We demonstrate that U-smile LE method outperforms AUC-based selection in imbalanced scenarios^2,5,7^.
3. We show how U-smile patterns evolve during stepwise selection, providing class-specific insight into the contribution of individual predictors.

## Materials and methods

### U-smile visualization and interpretation

**Fig. 1:**
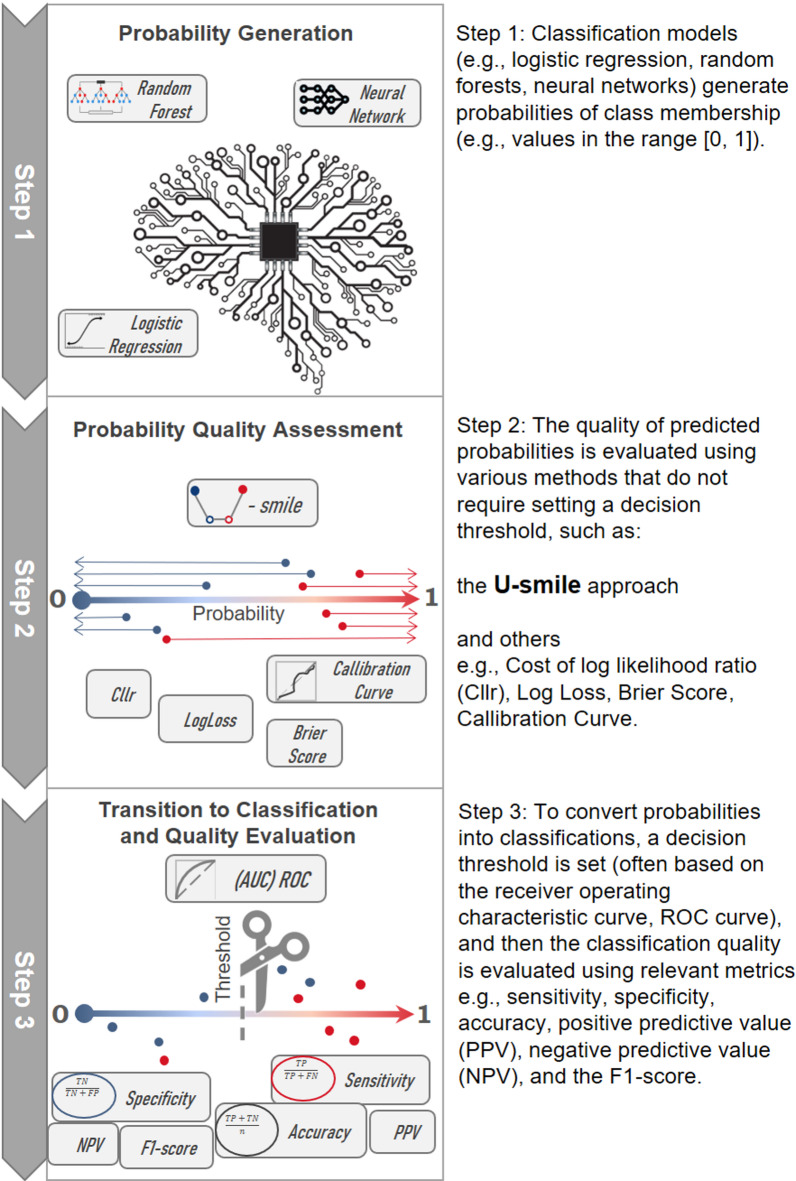
How does U-smile complement traditional metrics by offering class-specific evaluation already at the probability analysis stage?

Figure 1 shows how U-smile plots complement traditional evaluation methods for binary classification. It illustrates a typical workflow for developing and evaluating a probabilistic classification model, starting with probability generation, moving through quality assessment (where U-smile is used), and finally transitioning to discrete classifications and their evaluation.

**Fig. 2:**
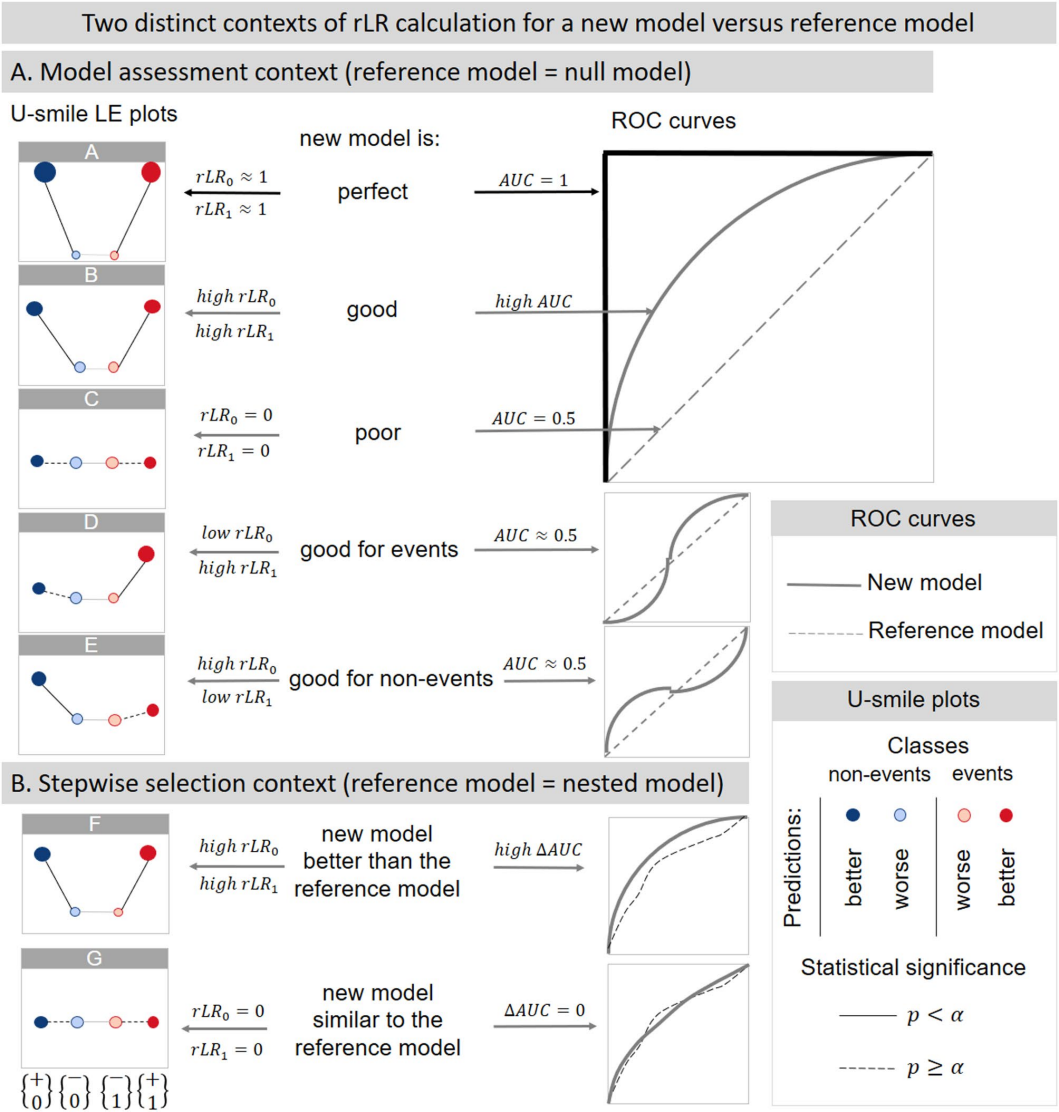
U-smile LE plots based on rLR coefficients and ROC curves – practical examples.

Figure 2 illustrates various U-smile LE patterns alongside their corresponding ROC curves, providing a comparative visualization of model performance. Each U-smile plot demonstrates the prediction quality of a new model in relation to a nested reference model (including the null model), while the paired ROC curves offer a traditional benchmarking perspective.

In general, the U-smile plot displays coefficients as circles – each subclass represented by different colors – corresponding to the subsets of samples where the model improved or worsened predictions relative to the reference. They are arranged along the X-axis in the order: $$\:\left\{\begin{array}{c}+\\\:0\end{array}\right\},\:\left\{\begin{array}{c}-\\\:0\end{array}\right\}\:,\:\left\{\begin{array}{c}-\\\:1\end{array}\right\}$$, $$\:\left\{\begin{array}{c}+\\\:1\end{array}\right\}$$,

where:

$$\:\left\{\begin{array}{c}+\\\:0\end{array}\right\},\:\left\{\begin{array}{c}-\\\:0\end{array}\right\}$$ present improvement/worsening prediction for non-event class,

$$\:\left\{\begin{array}{c}+\\\:1\end{array}\right\},\:\left\{\begin{array}{c}-\\\:1\end{array}\right\}$$ presents improvement/worsening prediction for event class.

We use the same operation for the U-smile LE method. The U-smile plot integrates three main visual components to provide a comprehensive assessment of a variable’s predictive contribution.

First, the vertical position of each point represents the rLR coefficient (see Sect.  2.2 for formal definition). Displayed on the Y-axis, the rLR quantifies the magnitude of prediction improvement or worsening for each subclass, providing the primary measure of a variable’s classification impact. The resulting pattern forms characteristic shapes that enable immediate visual interpretation: a pronounced symmetric ‘smile’ indicates strong predictive power for both classes, a flat line suggests no predictive power beyond the reference model, while an asymmetric smile reveals superior classification in only one class.

Second, as a configurable option, the point size corresponds to the I coefficient, which represents the proportion of individuals in each subclass^11^. This indicates how many individuals experience prediction changes, complementing the magnitude information provided by the rLR.

Third, we introduce statistical significance testing to the U-smile framework (detailed in Sect.  2.3). For parametric models, on the U-smile LE plot, solid lines represent statistically significant improvements (p < α), while dashed lines indicate non-significant changes (p ≥ α), providing immediate visual identification of which class-specific enhancements are statistically reliable.

A model that is both accurate and widely beneficial displays a deep ‘smile’ with high rLR values (large magnitude of improvement), large outer points (many individuals improved), small inner points (few individuals worsened), and solid lines indicating statistically significant improvements in both classes.

**Directionality of predictors and extreme performance cases**:

The U-smile LE method evaluates models based on predicted probabilities, irrespective of whether predictors are stimulants (predictors positively associated with the event class) or destimulants (predictors negatively associated with the event class). In a well-specified model, higher probabilities are correctly assigned to the true event class, yielding a high AUC and a symmetric U-smile.

In pathological cases where a model would perform worse than random (e.g., AUROC ≈ 0), the U-smile plot shows an inverted or highly asymmetric pattern, with negative rLR contributions. This illustrates how U-smile LE provides nuanced, class-stratified diagnostics even under extreme performance conditions.

### The U-smile LE method

#### Two distinct contexts of rLR calculation: (A) model assessment and (B) Stepwise selection

The U-smile LE method and its core metric, the rLR, can be applied in two distinct yet complementary contexts, which determine the choice of the reference model:

- A. Model assessment context: for the purpose of providing a standalone, absolute evaluation of a single model’s performance, the rLR is calculated using the null model as the reference. In this context, the rLR quantifies the model’s total predictive power, serving as an absolute measure of effectiveness. Figure 2 illustrates this comprehensive assessment for various models.
- B. Stepwise selection context: in the procedure of stepwise variable selection, the rLR is used to evaluate the marginal improvement offered by adding a new variable. Here, the reference model is the previous, nested model from the preceding step. In this context, the rLR measures the incremental gain in performance achieved by the new variable(s), guiding the selection process.

#### Mathematical definition of rLR coefficients

The following mathematical definitions underpin the relative Likelihood Ratio (rLR) calculation in both contexts. The U-smile LE method extends traditional likelihood ratio testing by decomposing the $\:rLR$ into class-specific components. The fundamental $\:rLR$ coefficient is defined as:

$$\:rLR=\frac{LR}{maxLR}$$

where $\:LR$ represents the likelihood ratio statistic comparing a new model to a reference model, and $\:maxLR$ represents the maximum achievable value if the new model is a perfect classifier.

The key innovation of the U-smile framework lies in its hierarchical decomposition, which we also employed in the U-smile LE method:

Level 3 (Overall):

$\:rLR$: Overall likelihood-based evaluation.

Level 2 (Class-specific):

$\:{rLR}_{0}$: Net coefficient for non-event class.

$\:{rLR}_{1}$: Net coefficient for event class.

$$\:r{LR}_{0}=\frac{{LR}_{0}}{max{LR}_{0}},\:\:\:\:r{LR}_{1}=\frac{{LR}_{1}}{max{LR}_{1}}$$

where:

$\:{LR}_{0}$ and $\:{LR}_{1}$ are class-specific likelihood ratio statistics,

$\:{maxLR}_{0}$ and $\:{maxLR}_{1}$ are class-specific maximum improvements over the reference model.

Level 1 (Subclass-specific):

$\:{rLR}_{0}^{+}$, $\:{rLR}_{0}^{-}$: Improvement/worsening for non-event class.

$\:{rLR}_{1}^{+}$, $\:{rLR}_{1}^{-}$: Improvement/worsening for event class.

$$\:{rLR}_{0}^{+}=\frac{{LR}_{0}^{+}}{max{LR}_{0}},\:\:\:\:{rLR}_{0}^{-}=\frac{{LR}_{0}^{-}}{max{LR}_{0}}{,\:\:\:\:\:\:\:\:rLR}_{1}^{+}=\frac{{LR}_{1}^{+}}{max{LR}_{1}},\:\:\:\:{rLR}_{0}^{-}=\frac{{LR}_{1}^{-}}{max{LR}_{1}}$$

#### The relations between the coefficients of the particular levels

$$\:LR={LR}_{0}+{LR}_{1}=\left({LR}_{0}^{+}-{LR}_{0}^{-}\right)+\left({LR}_{1}^{+}-{LR}_{1}^{-}\right)$$

$$\:{rLR}_{0}={rLR}_{0}^{+}-{rLR}_{0}^{-}$$

$$\:{rLR}_{1}={rLR}_{1}^{+}-{rLR}_{1}^{-}$$

$$\:rLR={rLR}_{0}\frac{max{LR}_{0}}{maxLR}+{rLR}_{1}\frac{max{LR}_{1}}{maxLR}$$

Mathematical details regarding the definition of the rLR for nested model comparison can be found in the Supplementary, Part A.

#### Relation to classical statistical measures

The rLR is conceptually linked to the likelihood-ratio principle and relates to McFadden’s pseudo-R² (Supplement, Part B), providing a statistically interpretable extension of classical goodness-of-fit measures^14^. This situates U-smile LE at the intersection of statistical inference and predictive modeling.

### Statistical significance testing

For parametric models, the $\:rLR$ statistic follows an asymptotic chi-squared distribution with k degrees of freedom, where k equals the difference in the number of variables between the compared models. The components $\:{rLR}_{0}$ and $\:{rLR}_{1}$, being linear transformations of $\:LR$, follow gamma distributions which enables separate significance testing for each class (see Supplementary, Part C for mathematical details).

In the U-smile LE plot, this class-specific significance is visualized using two distinct lines: one line connecting the blue points for the non-event class and one line connecting the red points for the event class. Solid lines indicate statistically significant improvements, while dashed lines indicate non-significant changes within each respective class.

The gray central line connecting the non-event and event classes is not currently used to display statistical test results. This design element is reserved for potential future extensions, such as direct statistical comparison between event and non-event classes.

### Experimental design

#### Synthetic data generation

We generated five synthetic data sets with different separation strengths:

1. low power: 2 informative features, 2 redundant features, high noise (10%);
2. medium power: 5 informative features, 2 redundant features, medium noise (5%);
3. high power: 8 informative features, no noise (0%);
4. imbalanced: medium power with 90/10 class distribution, majority class (non-event), minority class (event);
5. imbalanced and asymmetrical: imbalanced settings with variable V1 modified to be informative only for event class but not for non-event class (normal distributions: N(1,4) for non-event and N(0,1) for event).

Each dataset contained 1000 samples with 10 total features, of which the first 2–8 were truly informative depending on the scenario. However, the generation scenarios also included redundant variables which should not be selected in the final models, providing a more realistic test of the variable selection procedures.

#### Real-world clinical dataset

For validation in a practical setting, we used the Heart Disease dataset from the Cleveland Clinic Foundation^15,16^, available in the UCI Machine Learning Repository^17^. The binary classification task was to predict significant coronary artery disease (> 50% lumen stenosis in any major artery). The analysis included 661 subjects (347 healthy controls, 314 disease cases), representing a fairly good class balance.

The clinical variables used in our analysis were consistent with our previous work^11^ and included: age (in years), sex (1 = male, 0 = female), resting systolic blood pressure (bp, mmHg), serum cholesterol (chol, mg/dl), fasting blood glucose > 120 mg/dl (glu, 1 = yes, 0 = no), resting electrocardiographic changes (ecg, 0 = normal, 1 = ST-T abnormality, 2 = LVH), chest pain type (cp.), maximum heart rate during exercise (hr, bpm), exercise-induced angina (exang, 1 = yes, 0 = no), and exercise-induced ST depression (stde, mm).

This dataset provides a realistic clinical use-case where multiple predictor types are used for patient risk assessment.

#### Variable selection procedure

We implemented stepwise selection using four variants:

ROC: Traditional AUC maximization.

U-smile LE overall: overall $\:rLR$ improvement.

U-smile LE event: event-class $\:{rLR}_{1}$ improvement.

U-smile LE non-event: non-event class $\:{rLR}_{0}$ improvement.

Stepwise selection can be performed either through statistical significance testing – appropriate only for parametric models – or through effect-size criteria, which are applicable to any classifier. Because the goal of this study was not to compare logistic regression with random forests. Thus, to demonstrate that the U-smile LE method supports variable selection across different modeling paradigms, we used two selection schemes:

- For logistic regression, variable entry and removal were based on p-value thresholds (0.05 for entry, 0.10 for removal).
- For random forests (a non-parametric method without a meaningful likelihood-based significance test), selection was based on effect-size thresholds: a ≥ 3% improvement in AUC or ≥ 3% improvement in rLR.

Models in all selection procedures were built using 10-fold stratified cross-validation. Detailed model specifications and hyperparameters are provided in Supplement, Part D. The main text focuses on logistic regression results for interpretability, while random forest results are included in Supplement, Part E to illustrate the generalizability and robustness of the U-smile LE method across modeling frameworks.

#### Evaluation metrics

Model evaluation prioritized threshold-free metrics to enable direct comparison across models. The U-smile LE coefficients form the core of this framework, where $\:{rLR}_{1}$ serves as a threshold-free measure for event class assessment, and $\:{rLR}_{0}$ for non-event class evaluation, with overall rLR measuring global fit. These were complemented by AUC-ROC, AUC-PR and Cllr for comprehensive threshold-free assessment. Among threshold-dependent metrics, only F1-score is reported. The threshold-dependent metrics may not be directly comparable across models due to their calculation at model-specific cut-off points. Consequently, F1-score should be interpreted with caution, while primary conclusions rely on threshold-free metrics.

### Implementation

All analyzes were implemented in R (v4.2.2). Code and data for reproducing all experiments are available at [github.com/bbwieckowska/UsmileLE].

## Results

We built logistic regression and random forest models in a stepwise manner. The analysis was conducted for six data scenarios (low power, medium power, high power, imbalanced, imbalanced and asymmetrical, Heart Disease dataset) and four variable selection variants (ROC, U-smile LE overall, U-smile LE event, U-smile LE non-event). The results were evaluated in two distinct contexts: model assessment and stepwise selection procedure assessment.

Cross-validation ensured that all presented metrics were estimated on out-of-sample predictions (based on validation sets). For clarity, we present only the most significant findings in the main manuscript. Complete results for all combinations of data scenarios and selection variants are provided in the Supplementary Materials.

### Validation with logistic regression classifier

In Table 1 we present the results obtained from the imbalanced data scenario (90/10 class distribution).

**Table 1:** Evaluation of the predictive power of the logistic regression model in both unbalanced scenarios for all four variable selection procedures.

Scenario: Imbalanced				
Selection procedure	ROC	U-smile LE		
		Overall	Non-event	Event
Selected variables	V1, V6, V5	V1, V2, V3, V6	V1, V2, V3, V6	V1, V2, V3, V6
Number of variables	3	4	4	4
AUC-ROC	0.885	0.902	0.902	0.902
**AUC-PR**	**0.701**	**0.812**	**0.812**	**0.812**
Cllr	0.309	0.235	0.235	0.235
**F1-score**	**0.662**	**0.795**	**0.795**	**0.795**
$\:{rLR}_{0}$	0.384	0.566	0.566	0.566
$\:{rLR}_{1}$	0.465	0.560	0.560	0.560
$\:rLR$	0.348	0.579	0.579	0.579

Scenario: Imbalanced and asymmetrical				
Selection procedure	ROC	U-smile LE		
		Overall	Non-event	Event
Selected variables	V2, **V1**, V6, V5	V2, V7, V5, V4, **V1**	V2, V7, V5, V4	**V1**, V2, V4, V5, V6, V3
Number of variables	4	5	4	6
AUC-ROC	0.854	0.904	0.893	0.908
AUC-PR	0.494	0.803	0.802	0.809
Cllr	0.404	0.242	0.245	0.241
F1-score	0.446	0.809	0.772	0.803
$\:{rLR}_{0}$	0.139	0.551	0.557	0.553
$\:{rLR}_{1}$	0.306	0.555	0.544	0.558
$\:rLR$	0.254	0.554	0.548	0.556

AUC-ROC: Area Under the Receiver Operating Characteristic curve; a measure of discrimination, AUC-PR: Area Under the Precision-Recall curve, a measure of event class detection effectiveness, Cllr: log-likelihood ratio cost; a probabilistic loss metric, F1-score: balanced measure of event class accuracy (threshold-dependent), rLR: relative Likelihood Ratio; the core U-smile LE metric quantifying a model’s overall predictive improvement over a reference model, $\:{rLR}_{1}$: component of rLR quantifying predictive improvement specifically for the event class, $\:{rLR}_{0}$: component of rLR quantifying predictive improvement specifically for the non-event class.

The results in Table 1 compare variable selection methods across two imbalanced scenarios. The U-smile LE method has three variants, each with a different focus: one improves the event class, second the non-event class, and third aims for overall model improvement.

In the standard imbalanced scenario, all three U-smile LE variants selected the same set of four variables and demonstrated identical performance, which was superior to the ROC method across all key metrics. The U-smile LE performance metrics for minority class detection improved markedly: AUC-PR showed a 16% increase (0.701 → 0.812), while the F1-score exhibited a 21% relative increase (0.662 → 0.798).

The differences between the U-smile LE variants became clear in the asymmetrical scenario, where one variable (V1) was informative only for the event class. The variable selection variant focused on the non-event class correctly excluded this variable, as it was not useful for its specific goal. Despite selecting different variables in different U-smile LE variants, all final models significantly outperformed the ROC model. Their AUC-PR was over 60% higher (≥ 0.802 vs. 0.494) and their F1-score was over 70% higher (≥ 0.772 vs. 0.446).

In summary, the U-smile LE method consistently produced better models than the ROC method and demonstrated flexibility by adapting variable selection to specific predictive goals.

In balanced scenarios (low power, medium power, high power and Real Heart dataset), all methods performed comparably (Table 2).

**Table 2:** Evaluation of the predictive power of the logistic regression model in balanced and heart disease dataset scenarios, for all four variable selection procedures.

Scenario	Low power	Medium power		High power	Heart disease dataset		
Selection procedure	All methods	ROC	U-smile LE	All methods	ROC	U-smile LE	
						Overall/ event	Non-event
Selected Variables	**V1**,** V2**	**V1**,** V2**, V5	**V1**,** V2**, V3, V4	Various	cp., **stde**, **exang**, **sex**, age	cp., **stde**, **sex**, **exang**, age, chol, hr	cp., **exang**, **stde**, **sex**, glu
# Vars	2	3	4	4–5	5	7	5
AUC-ROC	0.947	0.978	0.980	~ 1.000	0.880	0.885	0.876
AUC-PR	0.924	0.976	0.976	~ 1.000	0.870	0.869	0.867
Cllr	0.351	0.272	0.225	0.000	0.635	0.622	0.638
F1-score	0.934	0.945	0.953	~ 1.000	0.771	0.806	0.785
rLR0	0.633	0.728	0.786	~ 1.000	0.371	0.376	0.373
rLR1	0.666	0.729	0.764	~ 1.000	0.360	0.378	0.350
rLR	0.650	0.729	0.775	~ 1.000	0.365	0.377	0.361

AUC-ROC: Area Under the Receiver Operating Characteristic curve; a measure of discrimination, AUC-PR: Area Under the Precision-Recall curve, a measure of event class detection effectiveness, Cllr: log-likelihood ratio cost; a probabilistic loss metric, F1-score: balanced measure of event class accuracy (threshold-dependent), rLR: relative Likelihood Ratio; the core U-smile LE metric quantifying a model’s overall predictive improvement over a reference model, $\:{rLR}_{1}$: component of rLR quantifying predictive improvement specifically for the event class, $\:{rLR}_{0}$: component of rLR quantifying predictive improvement specifically for the non-event class.

The results demonstrate that for balanced scenarios, including the Heart Disease dataset, the different variable selection procedures produced highly consistent outcomes. In the Low Power Scenario, all methods converged on the same two variables (V1, V2) and achieved identical performance metrics. The High Power Scenario further reinforced this trend, where all methods attained near-perfect results (~ 1.000) across all key metrics, regardless of the specific variables selected. This confirms that with very strong predictive signals, the differences between selection methods become marginal. Similar agreement continued in the Heart Disease dataset, where all methods showed comparable performance in AUC and AUC-PR. The U-smile LE methods exhibited only a marginal tendency to build slightly larger models, which corresponded to minimal improvements in the F1-score and a very balanced class-specific performance. The detailed results for each selection procedure are included in the Supplementary, part E.

### Evolution of U-smile patterns during variable selection for a logistic regression classifier

To illustrate the capabilities of the U-smile LE method in visualizing the stepwise variable selection and to compare them with the capabilities of the ROC curve and the PR curve, we focused on a representative subset of our comprehensive analysis. Rather than presenting 6 data scenarios, with 4 variable selection methods, and evaluating two distinct contexts, we selected one key scenario, with one representative variable selection procedure, and examined both evaluation contexts. Context A presents the evaluation of each model, while Context B presents the comparisons of the model built at a given step with the previous model.

**Fig. 3:**
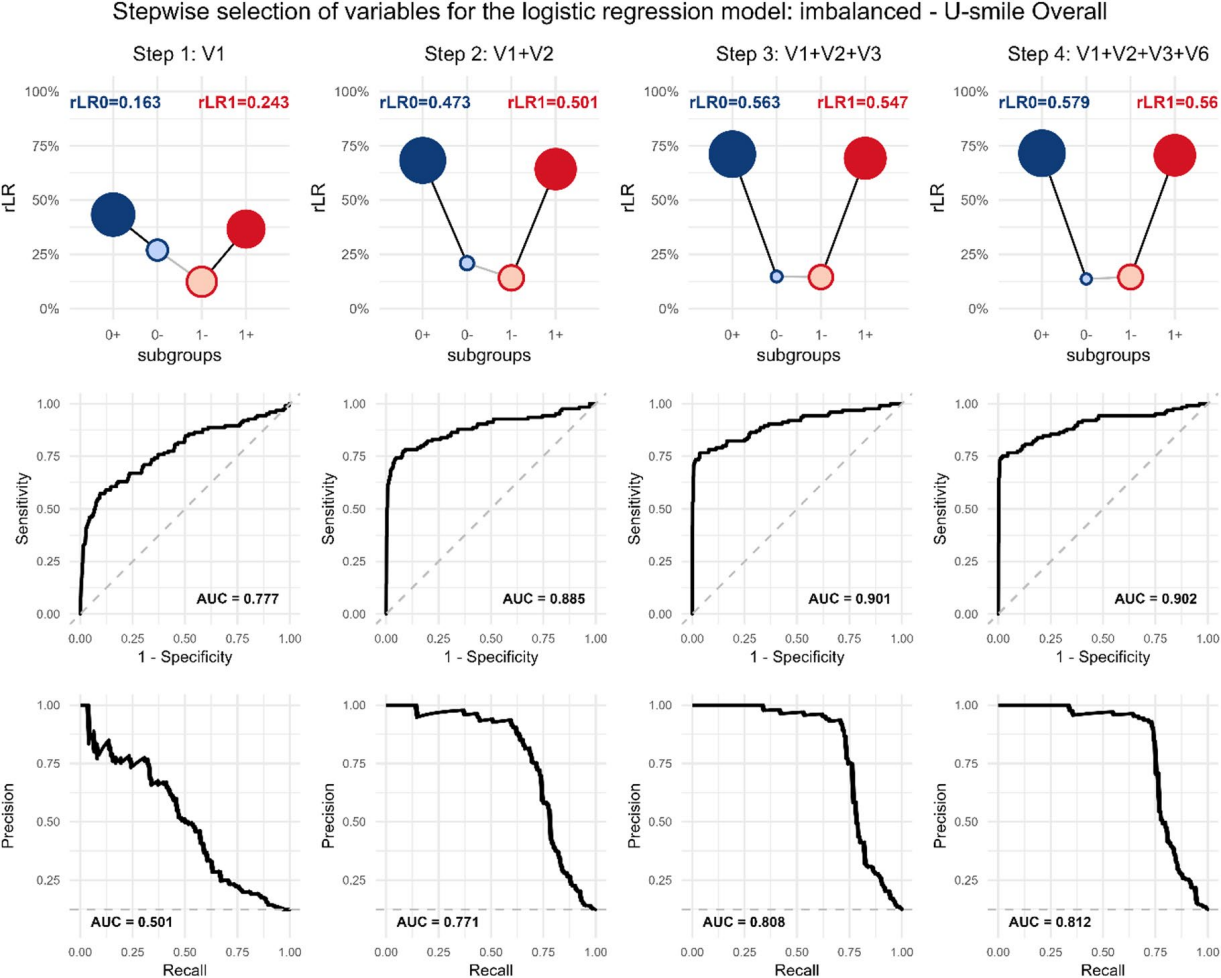
Model performance evolution during stepwise selection for logistic regression: model assessment (Context A: model assessment). Scenario: imbalanced, selection procedure: U-smile LE.

Figure 3 illustrates the stepwise evolution of U-smile plots during variable selection for the standard imbalanced scenario using the U-smile LE (Context A). This visualization provides unique insights into how the absolute predictive power for both classes improves cumulatively as variables are added.

When more variables are added, the sequences reveal a systematic progression toward symmetry:

Step 1 (V1 only): Asymmetric U-smile favoring event class ($\:{rLR}_{0}$*= 0.163*, $\:{rLR}_{1}$= 0.243).

Step 2 (V1, V2): Improved overall performance but maintained asymmetry.

Step 3 (V1, V2, V3): Near-perfect symmetry achieved ($\:{rLR}_{0}$*= 0.563*, $\:{rLR}_{1}$= 0.547).

Step 4 (V1, V2, V3, V6): Final statistically significant improvement delivers optimal performance ($\:{rLR}_{0}$*= 0.579*, $\:{rLR}_{1}$ = 0.560).

The sequence of U-smile (rLR) plots shows the performance of models developed at each step of the selection process, with each model (e.g., V1; V1 + V2; V1 + V2+V3) compared to the null model (Context A). The progressive deepening and symmetrization of the “smile”, along with the growth of the outer points, illustrate the cumulative improvement in both the magnitude and the prevalence of prediction improvements for both outcome classes as more variables are added.

The final U-smile plot shows remarkable symmetry (difference of only 0.019 between *rLR*_0_.

and $\:{rLR}_{1}$) despite the 90/10 class imbalance, indicating that the selected variable set provides balanced predictive power for both classes.

As the model evolves, the U-smile plot deepens and the outer points grow larger, indicating that the improvements affect a growing proportion of individuals. The lack of perfect symmetry in point sizes between non-event class and event class reflects the fact that a larger proportion of non-event class experience prediction improvement.

Figure 3 shows each model’s performance compared to a common baseline. However, stepwise selection works by directly comparing each new model to its immediate predecessor (e.g., a model with V1 + V2 vs. one with just V1).

**Fig. 4:**
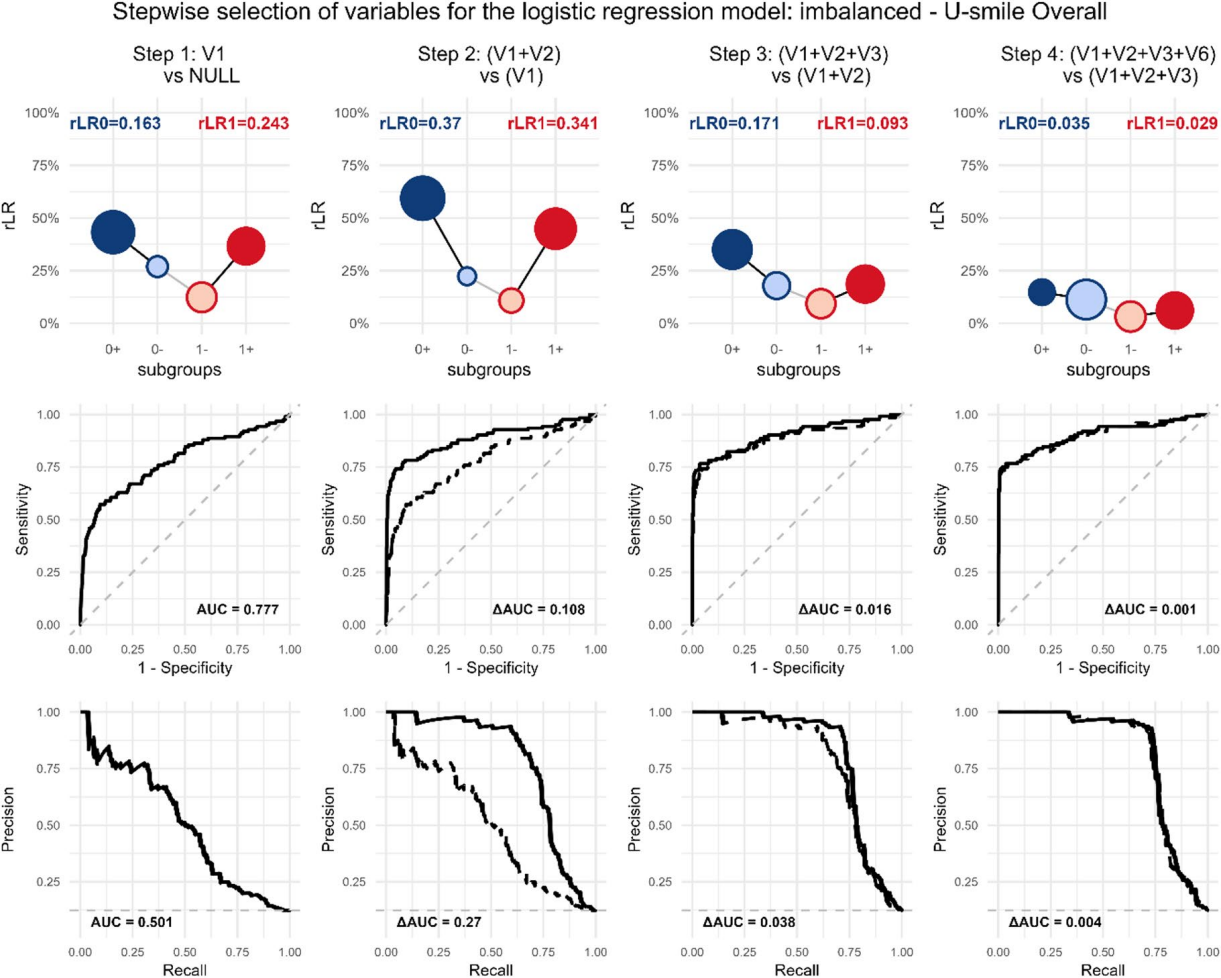
Incremental variable contribution in the stepwise selection process for logistic regression (Context B: nested model comparison). Scenario: imbalanced, selection procedure: U-smile LE.

Therefore, Fig. 4 provides an alternative visualization that highlights the improvement gained at each step (Context B). For example, it shows that adding variable V2 improves prediction for the non-event class more than for the event class. This ability to reveal *which class* benefits from a new variable is a key advantage of the U-smile LE method.

In contrast, while the ROC curves for these models also show an increasing AUC, they only offer a global measure of improved discrimination. Without a chosen cutoff, ROC analysis cannot indicate which specific class (event or non-event) improved.

### Validation with random forest classifier

We replicated the stepwise variable selection procedure using a random forest classifier to evaluate the U-smile LE method’s applicability to non-parametric models. Variable selection was based on effect-size thresholds (≥ 2% improvement in the relevant metric), as random forests do not provide p-values for traditional significance testing.

The complete numerical results for all scenarios are provided in Supplementary, Part E. In the imbalanced scenario (90/10 class distribution) using U-smile LE (overall) selection, the method selected five variables (V1, V2, V3, V5, V7). The final model achieved balanced predictive power for both classes (rLR₀ = 0.549, rLR₁ = 0.554) despite severe class imbalance.

**Fig. 5:**
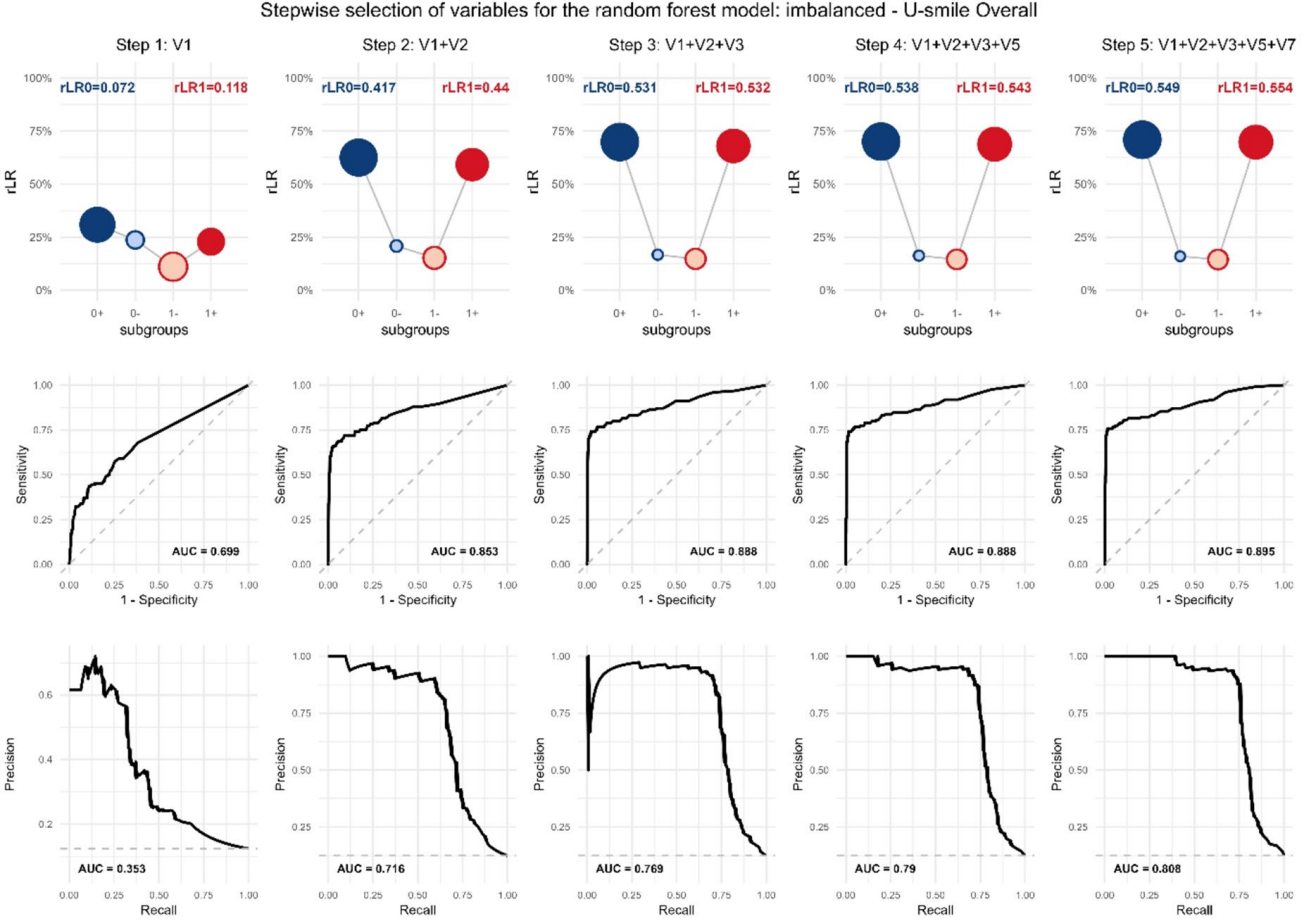
Model performance evolution during stepwise selection for random forest: model assessment (Context A: model assessment). Scenario: imbalanced, selection procedure: U-smile LE (overall).

Figure 5 shows the evolution of model performance in absolute terms (Context A: each model compared to the null model). The U-smile plots demonstrate cumulative improvement as variables are added, with the “smile” becoming progressively deeper and more symmetric.

**Fig. 6:**
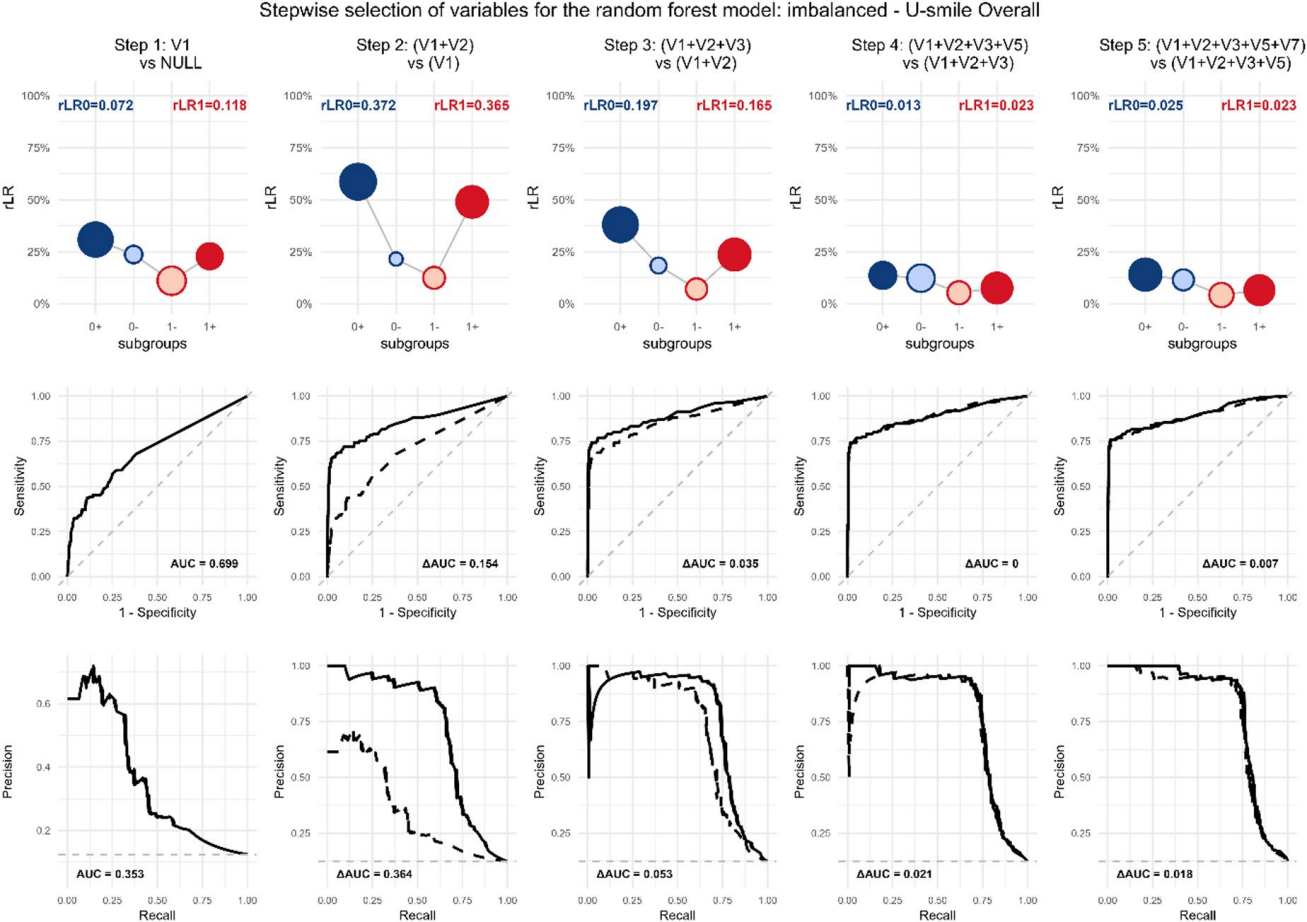
Incremental variable contribution in the stepwise selection process for random forest (Context B: nested model comparison). Scenario: imbalanced, selection procedure: U-smile LE (overall).

Figure 6 presents the incremental view of the selection process (Context B: each model compared to its immediate predecessor). This visualization reveals the class-specific contribution of each added variable. The corresponding ROC curves show increasing AUC but lack this class-stratified information.

The patterns observed with random forest were consistent with those from logistic regression, confirming the method’s consistency across different modeling approaches.

## Discussion

### Threshold-free evaluation

In this study, we focused on threshold-free metrics (rLR, AUC-ROC, AUC-PR, Cllr) to enable model comparison without setting an arbitrary decision threshold, which varies between models and contexts^3,5,6,18,19^. Sole reliance on threshold-dependent metrics like sensitivity or specificity can be misleading.

We included the F1-score as a widely used reference and for its utility in assessing minority class detection in our imbalanced data. However, our primary conclusions are based exclusively on threshold-free metrics.

### Class-specific value of evaluation

The key advantage of U-smile LE is its ability to evaluate the event and non-event classes separately, which is vital when the cost of misclassification differs^7,9^. While not solving all evaluation challenges, this separation provides crucial, intuitive insights. The method visualizes predictive quality directly: a deeper “smile” indicates better performance, and its symmetry reveals how equally both classes are modeled.

Our results suggest that such separation has practical significance. Some variables improved predictions mainly for the event class, resulting in asymmetry in the U-smile shape. This effect would be difficult to notice based on AUC alone, which aggregates information from both classes. U-smile LE does not replace classical metrics but constitutes a clear, intuitive complement – particularly when we care about the balance between classes or wish to explicitly assess their imbalance.

### Advantage in imbalanced scenarios

Our findings confirm that U-smile LE is particularly informative for imbalanced datasets. In the core 90/10 scenario, all its variants improved minority-class detection more effectively than ROC-based selection. They increased AUC-PR by 16% and the F1-score by 21%, whereas ROC metrics are known to be less discriminative under severe imbalance^5,20^. This aligns with recommendations to use alternative metrics like Precision-Recall curves and specialized methods for imbalanced data^21^.

The asymmetric variants of U-smile LE behaved as expected: the event-oriented version selected predictors informative for event class (boosting AUC-PR by 60% in the asymmetric scenario), whereas the non-event-oriented version appropriately excluded them. This demonstrates that rLR-based selection is sensitive to modeling objectives and maintains interpretability even under strong imbalance.

### Interpretability and model Understanding

U-smile LE aligns with Explainable AI principles by providing synthetic insights into predictive quality, though it is not a full model interpretation tool^22–24^. Its visual format – showing point positions and line types – clearly illustrates the impact of adding variables in a stepwise procedure.

This visualization proved especially valuable for monitoring class balance. For instance, in the imbalanced dataset, variables V1 and V2 primarily improved event-class predictions, V3 then restored balance, and V6 finalized a stable, symmetric model. Such nuanced class-level dynamics, difficult to capture using global metrics alone, highlight the added diagnostic value of the U-smile LE method.

### Model-agnostic applicability

The U-smile LE method is completely model-agnostic. We only evaluate the output probabilities – so any probabilistic algorithm can be evaluated identically. We tested two very different methods, parametric: logistic regression – stable, smooth, classical – and nonparametric: random forest – with more uncertain and less stable predictions^25,26^. The results showed that the U-smile LE method faithfully reflects the properties of both models. Variable selection requires only careful consideration of their assumptions; i.e., parametric methods can based on p-values ​​or effect sizes, whereas nonparametric methods based solely on effect sizes.

### Method limitations and future research directions

U-smile LE requires well-calibrated probabilities, which may necessitate additional calibration steps for complex or highly flexible models^27,28^. Moreover, rLR-based selection may favour more complex models, analogous to classical LR tests.

Future work may include multi-class extensions, integration with advanced calibration techniques, and exploration of penalised rLR versions (similar to the Akaike Information Criterion or Bayesian Information Criterion^29^) as well as automatic model selection procedures guided by rLR. Such extensions could also explore connections with advanced imbalance-aware evaluation approaches, like Precision-Recall-Gain curves^30^, to further enhance the interpretability of class-specific performance in highly skewed domains.

### Potential applications of the U-smile LE method

The U-smile LE method is a specific development within the wider U-smile framework, particularly well-suited for domains requiring interpretable, class-specific evaluation in imbalanced binary classification tasks. Its ability to visualize relative likelihood ratios separately for events (rLR1) and non-events (rLR0) offers a significant advantage over aggregate metrics like AUC, especially when the cost of misclassification differs between classes. In medicine and healthcare, this framework naturally supports scenarios where correctly detecting rare but critical cases is paramount. Applications include disease diagnosis, such as differentiating malignant from benign tumors; prognosis, for identifying patients at high risk of cardiovascular events; and treatment response, where prediction aids in selecting personalized therapies. By isolating performance on events, clinicians can verify that a new biomarker or risk score genuinely improves sensitivity to disease without compromising specificity for healthy individuals.

Beyond clinical settings, the U-smile framework holds substantial promise in sports science and analytics, where distinguishing exceptional outcomes from standard performance is essential. The method is invaluable for optimizing models in injury risk assessment—predicting rare occurrences, such as hamstring tears—and for identifying elite talent. A defining strength of the U-smile LE method is its compact visualization via U-smile plots, which effectively summarize complex likelihood metrics on constrained interfaces. This makes the method ideal for deployment in smartphone apps or smartwatches, allowing coaches and analysts to monitor shifting prediction trends and assess an athlete’s risk or performance potential in real-time simply by examining tiny U-smile plots.

The utility of the framework extends to other high-stakes fields characterized by class imbalance, including finance, energy, and industrial safety. In the financial sector, the method can enhance credit default prediction, fraud detection, and the forecasting of stock or currency movements by isolating improvements in detecting rare adverse events. Similarly, in energy forecasting, it aids in predicting critical shifts in production or consumption. In industrial settings, it can be used to forecast mechanical failures or aviation risks. Across all these domains, the U-smile framework provides a unified, interpretable lens for decision-making, ensuring that model updates via the LE method translate into actionable insights for both rare events and common outcomes.

## Key conclusions

U-smile LE method is a novel threshold-free approach for evaluating binary classifiers using the relative Likelihood Ratio (rLR), which quantifies predictive power without classification thresholds.

Unlike ROC curves, a single U-smile plot provides class-specific evaluation, separately assessing event and non-event classes without requiring a cutoff point.

The U-smile visualization offers complex interpretation:

- Quantitative assessment: smile height and shape (rLR).
- Qualitative insight: point size reflecting subgroup proportions.
- Statistical significance: line type (solid vs. dashed).

Class balance indication: symmetry reflects equal importance of both classes.

The method outperforms traditional variable selection under imbalance, enhancing minority class detection beyond AUC-based approaches.

As a model-agnostic tool, it extends beyond medicine to domains like sports analytics (e.g., injury and performance prediction), adding diagnostic value even in balanced data through class-stratified evaluation.

## Supplementary Information

Below is the link to the electronic supplementary material.

Supplementary Material 1

## Data Availability

We used the Heart Disease dataset [Detrano R, Janosi A, Steinbrunn W, Pfisterer M, Schmid J-J, Sandhu S, et al. International application of a new probability algorithm for the diagnosis of coronary artery disease. The American Journal of Cardiology 1989;64:304–10. https://doi.org/10.1016/0002-9149(89)90524-9.] from the public Machine Learning Repository [Aha D. UCI Machine Learning Repository: Heart Disease Data Set n.d. https://archive.ics.uci.edu/ml/datasets/heart+disease]. Random data similarly to the results presented in the paper can be generated again using the code on github.com/bbwieckowska/UsmileLE.
