# Supplementary material for: Usmile likelihood evaluation provides robust threshold free assessment of binary classification models for balanced and imbalanced datasets
Source: Sci Rep. 2026 Feb 20;16:10000. doi: 10.1038/s41598-026-40545-z (PMC13022492; doi:10.1038/s41598-026-40545-z)
Supplement: Supplementary file 1 — Supplementary Material 1 [file 41598_2026_40545_MOESM1_ESM.docx]

Usmile likelihood evaluation provides robust threshold free assessment of binary classification models for balanced and imbalanced datasets

B. Więckowska & P. Guzik

# Supplementary

## **(A) Definition of the relative Likelihood Ratio (rLR) for nested model comparison**

The relative likelihood ratio $(rLR)$ coefficient for comparing a new model to a smaller, nested reference model is defined as:

$$rLR=\frac{LR}{maxLR},$$

where:

- $LR$ is the likelihood ratio statistic comparing the new model to the null model,
- $maxLR$ represents the maximum achievable improvement over the reference model.

The likelihood ratio statistic (LR) is calculated as:

$LR=-2ln\left( \frac{L_{ref}}{L_{new}} \right)=-2\left[ ln\left( L_{ref} \right)-ln\left( L_{new} \right) \right]$,

where:

- $L_{ref}$ is the likelihood function of the reference (smaller) model,
- $L_{new}$ is the likelihood function of the new (larger) model.

The log-likelihood functions are given by:

$ln\left( L_{ref} \right)=\sum_{i=1}^{n} \left[ y_{i}ln\left( p_{i}^{ref} \right)+\left( 1-y_{i} \right)ln\left( 1-p_{i}^{ref} \right) \right],$

$ln\left( L_{new} \right)=\sum_{i=1}^{n} \left[ y_{i}ln\left( p_{i}^{new} \right)+\left( 1-y_{i} \right)ln\left( 1-p_{i}^{new} \right) \right]$,

where:

- $y_{i}\in\left\{ 0, 1 \right\}$ is the observed outcome (1 if the event occurred, 0 otherwise),
- $p_{i}^{ref}$ is the predicted probability from the reference model (if the smaller model is the null model, it is a constant, equal to the event frequency, $p_{i}^{ref} =\frac{\#1}{n}$, where #1 is the number of events and n is the sample size)
- $p_{i}^{new}$ is the predicted probability from the new model.

Maximum value of LR (maxLR) for nested model comparison

Derivation of the maximum value $(maxLR)$

- Log-likelihood of the reference model ($ln\left( L_{ref} \right)$):

For a given reference model and dataset, $ln\left( L_{ref} \right)=constant$.

- Log-likelihood of the new model ($ln\left( L_{new} \right)$):

The log-likelihood of the new model is always greater than or equal to that of the reference model:

$ln\left( L_{new} \right)\geq ln\left( L_{ref} \right)$.

Therefore:

$ln\left( L_{ref} \right)-ln\left( L_{new} \right)\leq0$.

- Likelihood ratio statistic ($LR$):

Multiplying the difference by -2 yields the likelihood ratio statistic, which is always non-negative:

$$LR=-2\left[ ln\left( L_{ref} \right)-ln\left( L_{new} \right) \right]\geq0$$

The larger the value of LR, the better the fit of the new model to the data.

- Maximum value of LR:

For binary models, the maximum theoretical value of the log-likelihood of the new model approaches zero ($ln\left( L_{new=perfect} \right) \to0$), indicating a perfect fit of the model to the data. In this case, the maximum value of the $LR$ statistic is:

$maxLR=-2\left[ ln\left( L_{ref} \right)-ln\left( L_{new=perfect} \right) \right]\underset{ln\left( L_{new=perfect} \right)=0}{=} -2ln\left( L_{ref} \right)$*.*

Since $ln(L_{ref})$ is a constant for a given reference model and dataset, $max LR$ is also a constant.

## **(B) Relationship between rLR coefficient and McFadden's R², when comparing a new model to the null model**

As we notice in Supplementary, Part A, since $maxLR=-2ln(L_{ref=null})$ (for the perfect model, where $ln\left( L_{new=perfect} \right) \to0$), the definition of rLR:

$$rLR=\frac{LR}{maxLR} \Rightarrow rLR=\frac{LR}{-2\ln\left( L_{ref=null} \right)}$$

Definition of McFadden's $R^{2}$:

$R_{McFaden}^{2}=1-\frac{ln(L_{new})}{\ln\left( L_{ref=null} \right)}$*.*

The LR statistic is related to the log-likelihoods of the models:

$LR=-2\left[ ln\left( L_{ref=null} \right)-ln(L_{new}) \right] \Rightarrow ln\left( L_{new} \right)= ln\left( L_{ref=null} \right)-\frac{LR}{2}$*.*

Substitution into McFadden's R²:

$R_{McFaden}^{2}=1-\frac{ln\left( L_{ref}=null \right)-\frac{LR}{2}}{\ln\left( L_{ref=null} \right)}=1-\left( 1-\frac{LR}{2\ln\left( L_{ref=null} \right)} \right)=\frac{LR}{2\ln\left( L_{ref=null} \right)}=rLR$*.*

Finally

$R_{McFaden}^{2}=rLR$*,*

where rLR is = McFadden's R²​ is scaled to the range [0, 1).

## **(C) Statistical properties and significance testing for nested models**

The rLR metric supports significance testing in parametric models. In a parametric model such as logistic regression, the likelihood ratio statistic follows the asymptotic χ² distribution (special case of gamma distribution), which allows for the calculation of a p-value.

Under the null hypothesis that the new model provides no improvement over the reference model, the likelihood ratio statistic follows:

$LR\sim{}_{k}^{2}\sim Gamma\left( shape=\frac{k}{2}, rate=\frac{1}{2} \right)$,

where k is the difference in the number of parameters between the new and reference models.

For class-specific components in nested model comparison, ${LR}_{0}$ and ${LR}_{1}$ can be derived as linear transformations of Gamma-distributed variable LR.

$X\sim Gamma\left( shape, rate \right)$ $\to$ $a\cdot X\sim Gamma\left( shape, \frac{rate}{a} \right)$.

Components transformation:

$$\begin{matrix} {LR}_{0}=c\cdot LR\sim Gamma\left( shape=\frac{k}{2}, rate=\frac{1}{2c} \right) \\ {LR}_{1}=\left( 1-c \right)\cdot LR\sim Gamma\left( shape=\frac{k}{2}, rate=\frac{1}{2\left( 1-c \right)} \right) \end{matrix}$$

where $n_{0}=c\cdot n$, $n_{1}=(1-c)\cdot n$, with $c\in\left( 0, 1 \right)$ being a fixed sample proportion (not a random variable like ${{LR}_{0}}/{LR}and {{LR}_{1}}/{LR}$)

Significance conditions:

For the full model:

p-value(LR) < α.

For the classes:

p-value(${LR}_{0}$) < α → significance in the non-event class (solid line on the plot),

p-value(${LR}_{0}$) < α → significance in the event class (solid line on the plot).

## **(D) Model assumptions and configuration**

This supplement details the technical assumptions, parameters, and implementation details for the logistic regression and random forest models used in the comparative analysis.

#### **1. Logistic regression**

**Package and function:**

- **Environment: R, package: stats (base R), function: glm() with family = binomial(link = "logit")**
- **Model configuration: distribution family: binomial, link function: logit, estimation Algorithm: iteratively re-weighted least squares (IWLS)**
- **convergence criteria: default settings of the stats package.**

**Variable selection process:**

- **Method:** bidirectional stepwise selection (forward and backward), **entry criterion:** p-value < 0.05, **removal criterion:** p-value > 0.10, **maximum variables:** 10
- **Validation:** 10-fold cross-validation with post-hoc calibration on the entire dataset. Predictions from the cross-validation were calibrated using a logistic regression model **(glm(labels ~ predictions, family = binomial)).**

#### **2. Random forest**

**Package and function:**

- Environment: R, package: randomForest, function: randomForest()
- Model Hyperparameters:
  The parameters were optimized for computational efficiency while maintaining predictive performance: number of trees (ntree): 250, number of variables sampled per split, mtry: floor(sqrt(number_of_predictors)), bootstrap sample size (sampsize): min(500, n_observations), minimum node size (nodesize): 10, sampling without replacement (replace): FALSE, maximum nodes (maxnodes): not restricted (left as default, NULL).

**Variable selection process:**

- **Method:** forward selection only, **entry Criterion:** effect size threshold of ≥2% improvement in the target metric (AUC, overall rLR, rLR for event class, or rLR for non-event class), **maximum variables:** 10
- **Validation:** 5-fold cross-validation with post-hoc calibration on the entire dataset, identical to the logistic regression procedure.

#### **3. General evaluation framework**

**Probability calibration:** For both models, raw probabilities obtained from cross-validation were calibrated on the entire dataset using a logistic regression calibration model: glm(labels ~ predictions, family = binomial). All reported probabilities and subsequent metrics are based on these calibrated values.

**Model improvement metrics (variable velection):**
The selection process used the following metrics to evaluate the improvement when adding or removing a variable:

- **AUC-ROC** (compared using DeLong's test)
- **U-smile Overall**
- **U-smile Event**
- **U-smile Nonevent**

**Implementation Notes:**

- A fixed random seed (set.seed(123)) was used throughout the analysis to ensure the reproducibility of results.
- For imbalanced class scenarios, a standard approach was applied without class weighting.
- The code was optimized for execution speed without compromising the core quality of the predictions.

## **(E) Validation results for logistic regression models and random forest**

Definition of Evaluation Metrics

The models were evaluated using the following set of threshold-free and threshold-dependent metrics:

**AUC-ROC (Area Under the Receiver Operating Characteristic Curve)**: Measures the overall ability of the model to discriminate between event class and non-event class across all possible classification thresholds. Values range from 0.5 (no discrimination, equivalent to random guessing) to 1.0 (perfect discrimination).

**AUC-PR (Area Under the Precision-Recall Curve)**: Particularly useful for imbalanced datasets, it evaluates the trade-off between precision (positive predictive value) and recall (sensitivity). Higher values indicate better performance, but its maximum value is influenced by class imbalance.

**Cllr (Cost of log Likelihood ratio)**: A threshold-free measure of calibration and discrimination derived from the log-likelihood. Lower values indicate better performance, with 0 representing a perfect model. Unlike other metrics, it has no upper bound for poor models.

**F1-score**: A threshold-dependent metric that is the harmonic mean of precision and recall. It ranges from 0 to 1, with 1 indicating perfect precision and recall. It is calculated at the model-specific optimal threshold (Youden index) and is most informative for the class of interest in imbalanced scenarios.

**rLR (relative Likelihood Ratio)**: The core, threshold-free metric of the U-smile LE method. It represents the overall model improvement over a reference model (e.g., the null model), normalized by the maximum possible improvement. It ranges from 0 (no improvement) to 1 (perfect prediction).

**rLR1 (net rLR for event class)**: The class-specific rLR coefficient quantifying the net prediction improvement for the event class.

**rLR0 (net rLR for non-event class)**: The class-specific rLR coefficient quantifying the net prediction improvement for the non-event class.

**I (Net Reclassification Index)**: The overall proportion of individuals in the dataset whose predictions were improved by the model. It ranges from 0 to 2.

**I1 (NRI for event class)**: The proportion of individuals from the event class whose predictions were improved.

**I0 (NRI for non-event class)**: The proportion of individuals from the non-event class whose predictions were improved.

**Comparative performance of logistic regression and random forest**

This section provides a detailed comparison of the variable selection outcomes between logistic regression and random forest classifiers across all experimental scenarios. The results demonstrate the consistent behaviour of the U-smile LE approach across different modeling paradigms.

**Balanced Scenarios (low, medium, high power)**
In balanced settings, both logistic regression and random forest models showed strong performance regardless of the selection method. For the low power scenario, all methods converged on the same 2-3 key variables (V1, V2, with random forest occasionally selecting V5), achieving nearly identical performance metrics (AUC-ROC: ~0.947-0.949). In the medium power scenario, U-smile methods tended to select slightly larger models (4 variables for logistic regression, 6-7 for random forest) compared to AUC-based selection (3 variables for both), with corresponding improvements in overall rLR (logistic regression: 0.728→0.775; random forest: 0.664→0.773). The high power scenario showed perfect or near-perfect classification for all methods with both classifiers.

**Table S.1.** Model performance and selected variables for logistic regression and random forest in balanced synthetic scenarios (low, medium, and high power).

| **model** | **logistic regression** | | | | **random forest** | | | |
| --- | --- | --- | --- | --- | --- | --- | --- | --- |
| **scenario** | **low** | | | | | | | |
| **method** | **AUC** | **U-smile overall** | **U-smile event** | **U-smile nonevent** | **AUC** | **U-smile overall** | **U-smile event** | **U-smile nonevent** |
| selected variables | V2, V1 | V2, V1 | V1, V2 | V1, V2 | V2, V3 | V2, V3, V5 | V2, V3, V5 | V2, V3, V5 |
| n variables | 2 | 2 | 2 | 2 | 2 | 3 | 3 | 3 |
| AUC-ROC | 0.947 | 0.947 | 0.947 | 0.947 | 0.942 | 0.949 | 0.949 | 0.949 |
| AUC-PR | 0.924 | 0.924 | 0.924 | 0.924 | 0.924 | 0.936 | 0.936 | 0.936 |
| Cllr | 0.351 | 0.351 | 0.351 | 0.351 | 0.363 | 0.349 | 0.349 | 0.349 |
| F1-score | 0.934 | 0.934 | 0.934 | 0.934 | 0.927 | 0.934 | 0.934 | 0.934 |
| rLR | 0.650 | 0.650 | 0.650 | 0.650 | 0.637 | 0.652 | 0.652 | 0.652 |
| rLR1 | 0.666 | 0.666 | 0.666 | 0.666 | 0.651 | 0.668 | 0.668 | 0.668 |
| rLR0 | 0.633 | 0.633 | 0.633 | 0.633 | 0.623 | 0.636 | 0.636 | 0.636 |
| I | 1.724 | 1.724 | 1.724 | 1.724 | 1.688 | 1.724 | 1.724 | 1.724 |
| I1 | 0.904 | 0.904 | 0.904 | 0.904 | 0.852 | 0.892 | 0.892 | 0.892 |
| I0 | 0.820 | 0.820 | 0.820 | 0.820 | 0.836 | 0.832 | 0.832 | 0.832 |
| **scenario** | **medium** | | | | | | | |
| selected variables | V1, V2, V5 | V1, V2, V3, V4 | V1, V2, V3, V4 | V1, V2, V3, V4 | V1, V2 | V1, V2, V3, V5, V7, V8, V4 | V1, V2, V3, V7, V5, V4 | V1, V2, V3, V4, V5, V7 |
| n variables | 3 | 4 | 4 | 4 | 2 | 7 | 6 | 6 |
| AUC-ROC | 0.978 | 0.980 | 0.980 | 0.980 | 0.962 | 0.979 | 0.980 | 0.981 |
| AUC-PR | 0.976 | 0.976 | 0.976 | 0.976 | 0.956 | 0.977 | 0.978 | 0.978 |
| Cllr | 0.272 | 0.225 | 0.225 | 0.225 | 0.336 | 0.227 | 0.227 | 0.225 |
| F1-score | 0.945 | 0.953 | 0.953 | 0.953 | 0.928 | 0.959 | 0.959 | 0.957 |
| rLR | 0.729 | 0.775 | 0.775 | 0.775 | 0.664 | 0.773 | 0.773 | 0.776 |
| rLR1 | 0.729 | 0.764 | 0.764 | 0.764 | 0.667 | 0.768 | 0.767 | 0.768 |
| rLR0 | 0.728 | 0.786 | 0.786 | 0.786 | 0.661 | 0.778 | 0.779 | 0.783 |
| I | 1.760 | 1.804 | 1.804 | 1.804 | 1.692 | 1.820 | 1.816 | 1.824 |
| I1 | 0.869 | 0.889 | 0.889 | 0.889 | 0.865 | 0.905 | 0.905 | 0.905 |
| I0 | 0.891 | 0.915 | 0.915 | 0.915 | 0.826 | 0.915 | 0.911 | 0.919 |
| **scenario** | **high** | | | | | | | |
| selected variables | V8, V4, V6, V7 | V8, V7, V2, V6, V5 | V1, V4, V7, V8, V2 | V1, V4, V7, V8, V2 | V8, V7 | V8, V7, V4, V10, V6, V2, V2, V2, V2, V2 | V8, V7, V4, V2, V5, V6 | V8, V7, V4, V10, V6, V2, V2, V2, V2, V2 |
| n variables | 4 | 5 | 5 | 5 | 2 | 10 | 6 | 10 |
| AUC-ROC | 1.000 | 1.000 | 1.000 | 1.000 | 1.000 | 1.000 | 1.000 | 1.000 |
| AUC-PR | 1.000 | 1.000 | 1.000 | 1.000 | 1.000 | 1.000 | 1.000 | 1.000 |
| Cllr | 0.000 | 0.000 | 0.000 | 0.000 | 0.041 | 0.000 | 0.000 | 0.000 |
| F1-score | 1.000 | 1.000 | 1.000 | 1.000 | 0.993 | 1.000 | 1.000 | 1.000 |
| rLR | 1.000 | 1.000 | 1.000 | 1.000 | 0.959 | 1.000 | 1.000 | 1.000 |
| rLR1 | 1.000 | 1.000 | 1.000 | 1.000 | 0.958 | 1.000 | 1.000 | 1.000 |
| rLR0 | 1.000 | 1.000 | 1.000 | 1.000 | 0.961 | 1.000 | 1.000 | 1.000 |
| I | 2.000 | 2.000 | 2.000 | 2.000 | 1.964 | 2.000 | 2.000 | 2.000 |
| I1 | 1.000 | 1.000 | 1.000 | 1.000 | 0.984 | 1.000 | 1.000 | 1.000 |
| I0 | 1.000 | 1.000 | 1.000 | 1.000 | 0.980 | 1.000 | 1.000 | 1.000 |

**Imbalanced Scenario (90/10)**
The imbalanced scenario revealed clear advantages for U-smile LE selection. For logistic regression, U-smile methods selected 4 variables (V1, V2, V3, V6) compared to 3 variables (V1, V6, V5) for AUC selection, resulting in substantial improvements: AUC-PR increased from 0.701 to 0.812 (+16%), F1-score from 0.662 to 0.795 (+20%), and overall rLR from 0.429 to 0.566 (+32%). The random forest models showed a similar pattern, with U-smile selection choosing 5 variables (V1, V2, V3, V5, V7) versus 3 for AUC selection, achieving better balanced performance (rLR₀=0.549, rLR₁=0.554) compared to the asymmetric AUC-selected model (rLR₀=0.531, rLR₁=0.532).

**Table s.2.** Model performance and selected variables for logistic regression and random forest in the imbalanced synthetic scenario (90/10 class distribution).

| **model** | **logistic regression** | | | | **random forest** | | | |
| --- | --- | --- | --- | --- | --- | --- | --- | --- |
| **scenario** | **imbalanced** | | | | | | | |
| **method** | **AUC** | **U-smile overall** | **U-smile event** | **U-smile nonevent** | **AUC** | **U-smile overall** | **U-smile event** | **U-smile nonevent** |
| selected variables | V1, V6, V5 | V1, V2, V3, V6 | V1, V2, V3, V6 | V1, V2, V3, V6 | V1, V2, V3 | V1, V2, V3, V5, V7 | V1, V2, V3, V5, V7 | V1, V2, V4 |
| n variables | 3 | 4 | 4 | 4 | 3 | 5 | 5 | 3 |
| AUC-ROC | 0.885 | 0.902 | 0.902 | 0.902 | 0.888 | 0.895 | 0.895 | 0.880 |
| AUC-PR | 0.701 | 0.812 | 0.812 | 0.812 | 0.769 | 0.808 | 0.808 | 0.767 |
| Cllr | 0.309 | 0.235 | 0.235 | 0.235 | 0.254 | 0.243 | 0.243 | 0.265 |
| F1-score | 0.662 | 0.795 | 0.795 | 0.795 | 0.780 | 0.814 | 0.814 | 0.781 |
| rLR | 0.429 | 0.566 | 0.566 | 0.566 | 0.532 | 0.552 | 0.552 | 0.511 |
| rLR1 | 0.465 | 0.560 | 0.560 | 0.560 | 0.532 | 0.554 | 0.554 | 0.505 |
| rLR0 | 0.348 | 0.579 | 0.579 | 0.579 | 0.531 | 0.549 | 0.549 | 0.523 |
| I | 1.361 | 1.441 | 1.441 | 1.441 | 1.386 | 1.429 | 1.429 | 1.328 |
| I1 | 0.532 | 0.500 | 0.500 | 0.500 | 0.484 | 0.516 | 0.516 | 0.435 |
| I0 | 0.829 | 0.941 | 0.941 | 0.941 | 0.902 | 0.913 | 0.913 | 0.893 |

**Imbalanced and Asymmetrical Scenario**
This challenging scenario highlighted the adaptive nature of U-smile LE. For logistic regression, the U-smile Event variant selected 6 variables including V1 (which was informative only for event class), while U-smile Nonevent correctly excluded V1, selecting only 4 variables. All U-smile variants substantially outperformed AUC selection, with AUC-PR improvements from 0.494 to ≥0.802 (>62% increase). For RF, the U-smile Event variant selected 7 variables and achieved the best event-class performance (AUC-PR=0.740), while both AUC and U-smile Nonevent methods failed to identify any informative variables in some runs, reflecting the instability of random forest in this challenging setting.

**Table S.3.** Model performance and selected variables logistic regression and random forest in the imbalanced and asymmetrical synthetic scenario. Note: For random forest, the nonevent variant did not select any variables.

| **model** | **logistic regression** | | | | **radom forest** | | | |
| --- | --- | --- | --- | --- | --- | --- | --- | --- |
| **scenario** | **imbalanced and asymmetrical** | | | | | | | |
| **method** | **AUC** | **U-smile overall** | **U-smile event** | **U-smile nonevent** | **AUC** | **U-smile overall** | **U-smile event** | **U-smile nonevent** |
| selected variables | V2, V1, V6, V5 | V2, V7, V5, V4, V1 | V1, V2, V4, V5, V6, V3 | V2, V7, V5, V4 | V2, V1, V6, V4 | none | V10, V1, V2, V7, V5, V3, V4 | none |
| n variables | 4 | 5 | 6 | 4 | 4 | 0 | 7 | 0 |
| AUC-ROC | 0.854 | 0.904 | 0.908 | 0.893 | 0.853 | - | 0.894 | - |
| AUC-PR | 0.494 | 0.803 | 0.809 | 0.802 | 0.556 | - | 0.740 | - |
| Cllr | 0.404 | 0.242 | 0.241 | 0.245 | 0.389 | - | 0.295 | - |
| F1-score | 0.446 | 0.809 | 0.803 | 0.772 | 0.512 | - | 0.620 | - |
| rLR | 0.254 | 0.554 | 0.556 | 0.548 | 0.283 | - | 0.456 |  |
| rLR1 | 0.306 | 0.555 | 0.558 | 0.544 | 0.325 | - | 0.477 | - |
| rLR0 | 0.139 | 0.551 | 0.553 | 0.557 | 0.190 | - | 0.410 | - |
| I | 1.033 | 1.457 | 1.438 | 1.429 | 1.078 | - | 1.317 | - |
| I1 | 0.339 | 0.516 | 0.484 | 0.500 | 0.339 | - | 0.500 | - |
| I0 | 0.694 | 0.941 | 0.954 | 0.929 | 0.740 | - | 0.817 | - |

**Real-World Heart Data**
Both classifiers showed consistent performance on the clinical dataset. U-smile methods tended to select slightly larger models (5-7 variables) compared to AUC selection (3-5 variables), with minimal differences in AUC-ROC and AUC-PR but modest improvements in F1-score and class balance. The random forest models generally achieved slightly higher performance metrics than logistic regression across all selection methods.

**Table S.4.** Model performance and selected variables for logistic regression and random forest on the real-world Heart Disease dataset.

| **model** | **logistic regression** | | | | **random forest** | | | |
| --- | --- | --- | --- | --- | --- | --- | --- | --- |
| **scenario** | **Heart Disease dataset** | | | | | | | |
| **method** | **AUC** | **U-smile overall** | **U-smile event** | **U-smile nonevent** | **AUC** | **U-smile overall** | **U-smile event** | **U-smile nonevent** |
| selected variables | cp, stde, exang, sex, age | cp, stde, sex, exang, age, chol, hr | cp, stde, sex, exang, age, chol, hr | cp, exang, stde, sex, glu | cp, stde, exang | cp, exang, sex | cp, exang, sex, age, bp, ecg | exang, cp, sex |
| n variables | 5 | 7 | 7 | 5 | 3 | 3 | 6 | 3 |
| AUC-ROC | 0.880 | 0.885 | 0.885 | 0.876 | 0.847 | 0.853 | 0.861 | 0.846 |
| AUC-PR | 0.870 | 0.869 | 0.869 | 0.867 | 0.830 | 0.837 | 0.830 | 0.821 |
| Cllr | 0.635 | 0.622 | 0.622 | 0.638 | 0.704 | 0.699 | 0.676 | 0.697 |
| F1-score | 0.771 | 0.806 | 0.806 | 0.785 | 0.734 | 0.776 | 0.790 | 0.777 |
| rLR | 0.365 | 0.377 | 0.377 | 0.361 | 0.295 | 0.301 | 0.324 | 0.302 |
| rLR1 | 0.360 | 0.378 | 0.378 | 0.350 | 0.285 | 0.295 | 0.342 | 0.297 |
| rLR0 | 0.371 | 0.376 | 0.376 | 0.373 | 0.306 | 0.306 | 0.304 | 0.308 |
| I | 1.157 | 1.187 | 1.187 | 1.123 | 1.099 | 1.100 | 1.128 | 1.102 |
| I1 | 0.561 | 0.573 | 0.573 | 0.510 | 0.433 | 0.567 | 0.561 | 0.586 |
| I0 | 0.597 | 0.614 | 0.614 | 0.614 | 0.666 | 0.533 | 0.568 | 0.516 |

**Model-Specific Patterns**
Random forest models consistently selected more variables than logistic regression across scenarios, reflecting the method's ability to capture complex interactions. However, this came with increased variability in selection stability, particularly in the asymmetrical scenario. Both classifiers benefited from U-smile LE guidance, with the approach providing consistent performance improvements in imbalanced settings regardless of the underlying algorithm.

The complete numerical results confirm that U-smile LE provides robust variable selection guidance across different classifier types, with particularly pronounced benefits in imbalanced classification scenarios where traditional AUC-based selection may lead to suboptimal model performance.
